# Production of ethanol from Jerusalem artichoke by mycelial pellets

**DOI:** 10.1038/s41598-019-55117-7

**Published:** 2019-12-06

**Authors:** Chao Zhang, Daoji Wu, Hongqi Yang, Huixue Ren

**Affiliations:** 1 School of Municipal and Environmental Engineering, Shandong Jianzhu University, JiNan, 250101 China; 2 Co-Innovation Center of Green Building, JiNan, 250101 China; 3 School of foreign languages and literature, Nanjing Tech University, Nanjing, 210009 China

**Keywords:** Applied microbiology, Industrial microbiology

## Abstract

Mycelial pellets formed by *Aspergillus niger* A-15 were used to immobilize the ethanol producing yeast *Saccharomyces cerevisiae* C-15. The operation parameters, such as agitation speed, temperature and mixed proportion of strains were studied. The optimal adsorption 66.9% was obtained when speed was 80r/min, temperature was 40 °C and mixed proportion(mycelial pellets: yeasts) was 1:10. With Jerusalem artichoke flour as substrate, 12.8% (V/V) of ethanol was obtained after 48 h by simultaneous saccharification and fermentation using mycelial pellets. And mycelial pellets could tolerate 19% (volume fraction) ethanol. The above results proved that this new technology was feasible, and it had the advantages of higher ethanol yield, long service life, repeated use, easy operation and lower cost in producing ethanol.

## Introduction

Corn is the major feedstock for the fuel ethanol production in China. However, this process consumes a large amount of corn, which poses a threat to national food security^1–5^. Jerusalem artichoke (JA) can be suitable for ethanol production as it contains 11–20% (w/w) carbohydrate of which 70–90% is inulin. Inulin, a fructan-type polysaccharide, consists of (2 → 1) linked β-D-fructosyl residues (*n* = 2–60), usually with an (1 ↔ 2) α-D-glucose end group. Inulin as feedstock for ethanol production is more advantageous than conventional material, such as starch^6–8^. For example, fermentable sugars can be obtained by enzymatic hydrolysis of inulin directly, which has the characteristics of simple process without high temperature liquefaction and low energy consumption. Compared with crop straw which is the representative of lignocellulosic biomass, ethanol production by inulin fermentation is easier to industrialize^9–11^. In addition, JA has the advantages of the high carbohydrate yields (5 and 14 ton/ha) and large-scale cultivation. This plant can be adapted to different agriculture conditions, because of the cold and drought tolerance, wind and sand resistance, saline tolerance, strong fecundity, and high pest and disease resistance^12,13^. Therefore, JA can be optimal non-grain feedstock for ethanol production. And there are many studies focused on producing ethanol using JA^12–14^.

Yeasts are frequently used in the process of ethanol production using JA as feedstock. However, yeast cannot secrete inulinase, and so inulin should be saccharified firstly. The most commonly used technology for the production of ethanol from JA is simultaneous saccharification and fermentation. Simultaneous saccharification and fermentation can convert the fermentable sugars from hydrolysate of inulin to ethanol simultaneously, preventing sugar accumulation which inhibits inulinase secretion, avoiding substrate inhibition, preventing bacteria pollution effectively, increasing ethanol yield. However, there are many shortcomings in this technology, such as lower ethanol yield and lower utilization rate of inulin^15–19^. In addition, there is a problem that strains cannot be reused, resulting in high production cost and environmental pollution. Cell immobilization technique can be used to solve the above problems. Immobilized yeasts can improve the efficiency of enzymatic reaction, so it has a good application prospect in the process of ethanol production. For example, Xue *et al*.^20,21^ reported that high-titer butanol production was observed with *Clostridium acetobutylicum* JB200 in a fibrous bed bioreactor. But the high cost of immobilization restricts its applications^22–26^. And most *Saccharomyces cerevisiae* can not directly utilize inulin, immobilization operation needs to fix a variety of strains, which will further increase the cost of immobilization. Furthermore, many investigations were focused on finding the high ethanol tolerance strains for ethanol production and relieving the inhibition of high concentration ethanol^27–30^.

Aiming at resolving the deficiency of the conventional technology, this study put forward a kind of carrier fermentation technology. At present, there are no reports of simultaneous saccharification and fermentation which are combined with immobilize carrier to producing ethanol. These two techniques are generally used separately, and there are no relevant reports about using immobilized pellets as fermenting microbe. This is the first time that this concept and technology are adopted to combine carrier with fermenting microbe in ethanol production. Because *A. niger* A-15 could secrete inulinase and form mycelial pellets, *A. niger* A-15 was used as carrier. As saccharification was required before fermentation, mycelial pellets formed by *A. niger* A-15 were used to immobilize the ethanol producing yeast *S. cerevisiae* C-15. Ethanol was produced by mycelial pellets through simultaneous saccharification and fermentation, to simplify the process and improve the ethanol tolerance and ethanol yield of yeast.

## Results

### Effect of temperature on the adsorption rate of yeast

**Figure 1:**
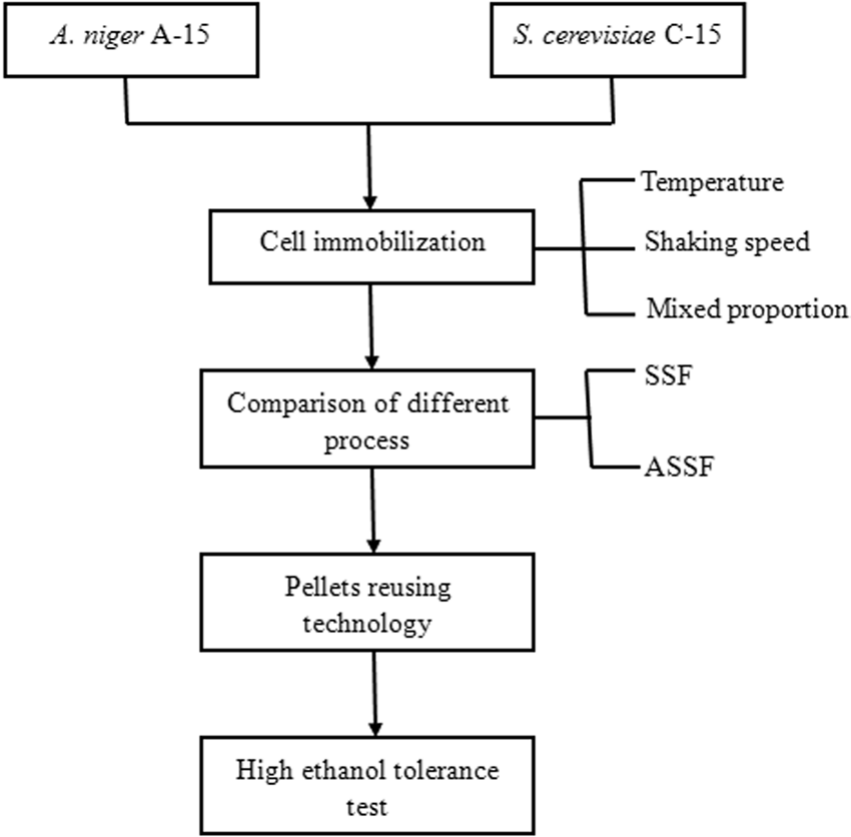
Flow chart of ASSF.

**Figure 2:**
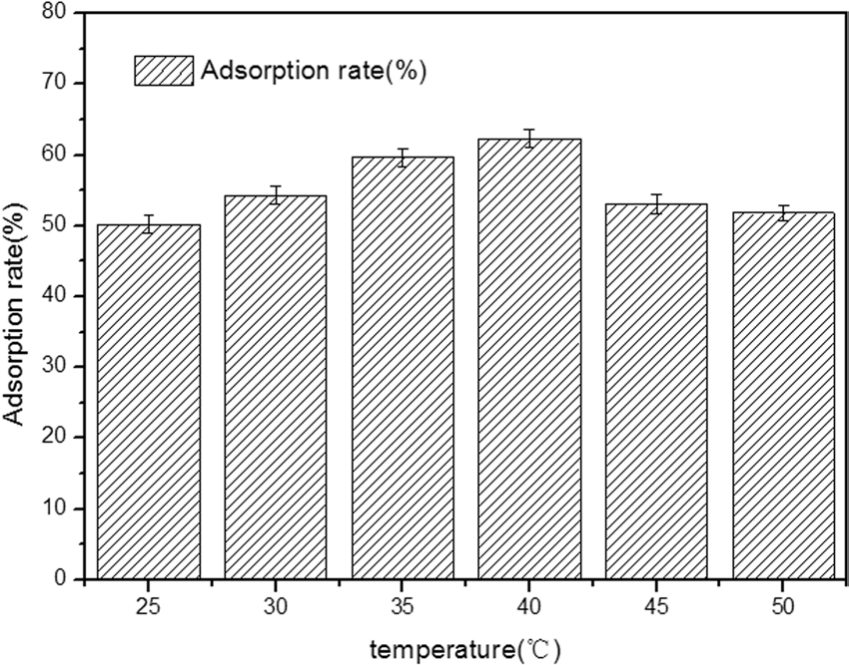
Effect of temperature on the adsorption rate of yeast.

The process diagram of the whole experiment is shown in Fig. 1.To determine how different temperatures affected the adsorption rate of yeast, the pellets of *A. niger* A-15 were conducted in 500 ml flask containing 100 mL yeast suspension. Then the yeast cells were adsorbed on a rotary shaker operating at different temperatures (25, 30, 35, 40, 45, 50 °C) for 2 h, shown in Fig. 2. The maximum adsorption rate of yeast was about 62.3 ± 0.1% at 40 °C. Therefore, temperature 40 °C was employed in the following experiments.

### Effect of shaking speed on the adsorption rate of yeast

**Figure 3:**
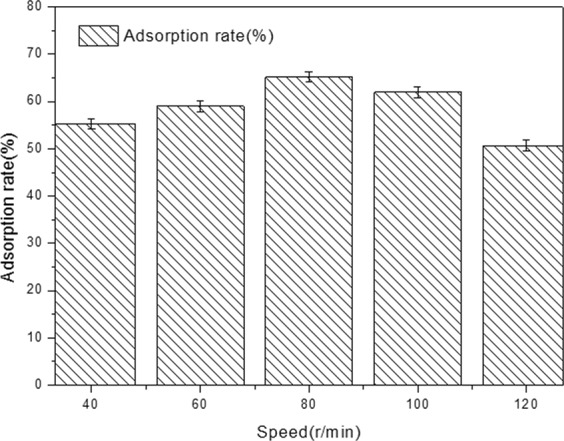
Effect of speed on the adsorption rate of yeast.

To determine how different shaking speeds affected the adsorption rate of yeast, the pellets of *A. niger* A-15 were conducted in 500 mL flask containing 100 mL yeast suspension. Then the yeast cells were adsorbed on a rotary shaker operating at different speeds (40, 60, 80, 100, 120 r/min) for 2 h, and the results are shown in Fig. 3. The maximum adsorption rate of yeast was about 65.1 ± 0.1% at 80 r/min. Therefore, the shaking speed 80 r/min was employed in the following experiments.

### Effect of mixed proportion on the adsorption rate of yeast

**Figure 4:**
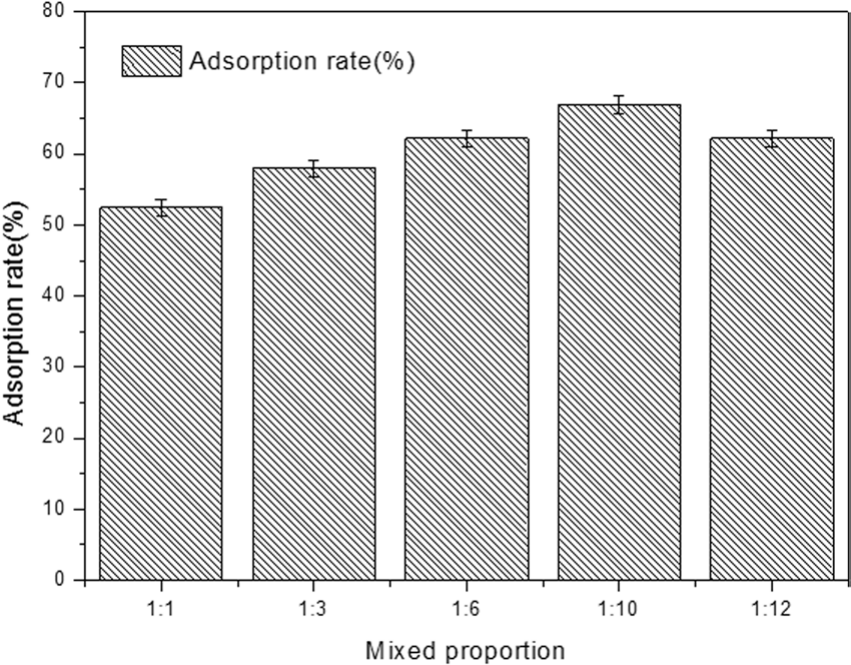
Effect of mixed proportion on the adsorption rate of yeast.

The effect of mixed proportion mycelial pellets: yeasts (1:1, 1:3, 1:6, 1:10, 1:12) on the adsorption rate of yeast were investigated, and the results are shown in Fig. 4. The mixed proportion refers to the ratio between the number of mycelial pellets and yeast.

As shown in Fig. 4, the proportion of strains could affect the formation of mixed mycelial pellets. The adsorption rate of pellets increased with the concentration of yeast. The maximum adsorption rate of yeast was about 66.9 ± 0.1% at 1:10. Any further increase in the concentration of yeast resulted in a decrease of the adsorption rate of pellets. This decrease-trend at high yeast concentration could be partly attributed to a result of no redundant adsorption sites on mycelial pellets.

**Figure 5:**
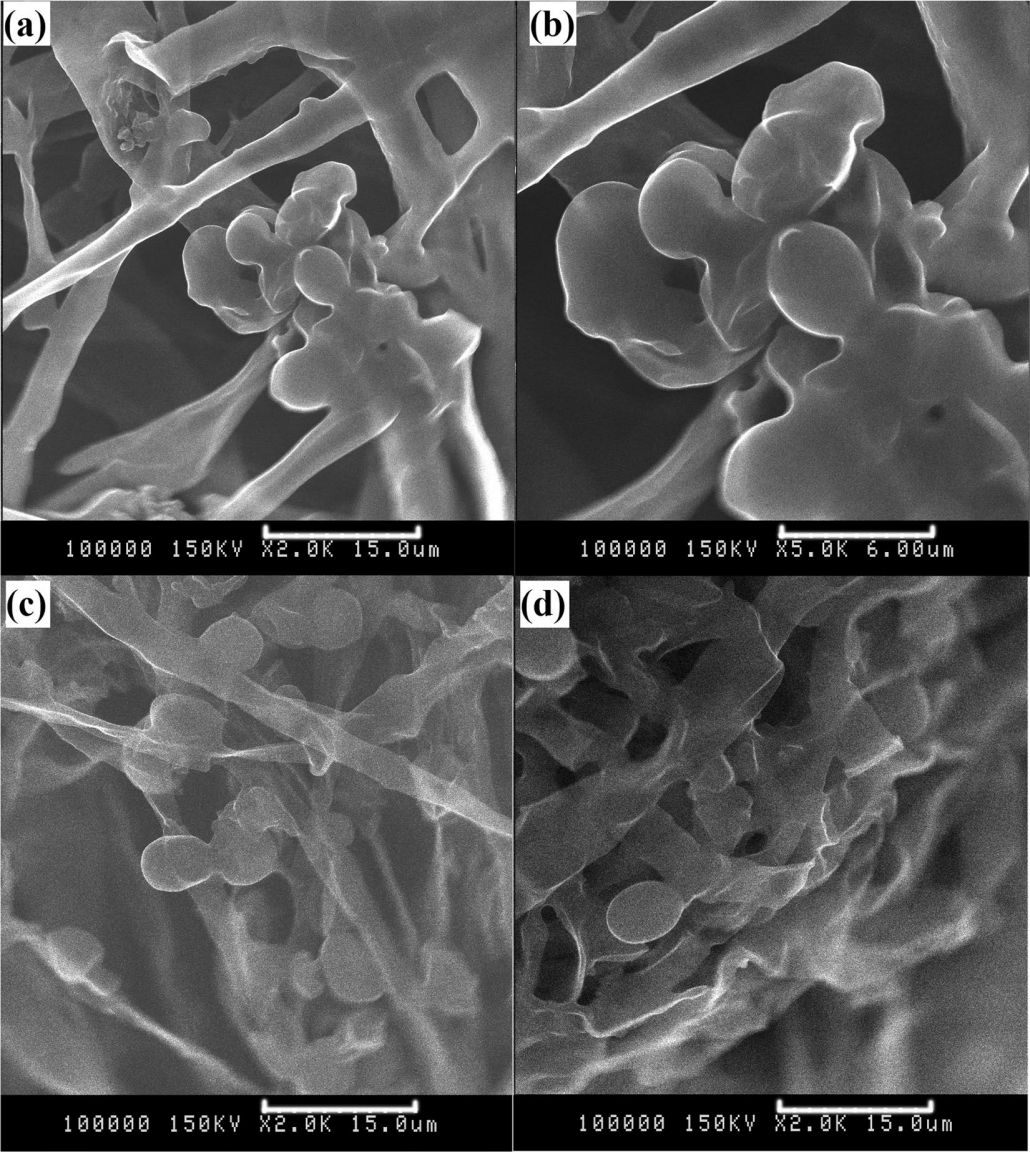
Adsorption of yeasts by mycelia pellets. (**a**,**b**) adsorption of yeasts by mycelia pellets (different magnification); (**c**) adsorption of yeasts by mycelia pellets (first recycle); (**d**) adsorption of yeasts by mycelia pellets (tenth recycle).

In addition to the electrostatic adsorption of surface charges, mycelial pellets also secrete a kind of extracellular polymer film on the surface of mycelia, which is mainly composed of polysaccharides and proteins. It makes the surface of mycelial pellets have a greater adhesion force, and is more favorable for yeast adsorption than other biological carriers^32^. It can be seen from Fig. 5(a,b) that yeast is adsorbed to mycelia in large quantities. In addition, unlike the microporous structure of particulate activated carbon, the inner space of mycelial pellets is larger, which is conducive to the entry and adsorption of yeast.

### Comparison of different fermentation modes

A bulk of literature has investigated the SSF process using Jerusalem artichoke as substrate for ethanol production^16–19^. However, as a new process, the feasibility and advantages of ASSF process were studied first in this study. Moreover, many processes in the literature use different strains, so it is impossible to compare these processes with ASSF under the same conditions. Therefore, this study chose a more representative process reported in literature 11 to compare with ASSF. Table 1 indicates the comparison of different fermentation modes. After 48 h cultivation, the ethanol yield by the pellets was approximately 1.19 times higher than that by conventional simultaneous saccharification and fermentation (12.8% versus 10.8%), and lower residual sugar concentration (5.8 g/L versus 1.5 g/L). Considering the ethanol yield and residual sugar concentration, carrier fermentation was much more economical. Moreover, the chemical oxygen demand (COD) of effluent showed that the COD of effluent produced after the ethanol distillation of the fermentation liquid was reduced from 54612 mg/L to 27641 mg/L of SSF, which is beneficial to reducing the pollution from the source.

**Table 1:** Comparison of different fermentation modes.

Mode	Ethanol concentration(%)	Ethanol yield(%)	Residual sugar (g/L)	Inulinase activity (U/mL)	COD of effluent (mg/L)	Remarks	Reference
SSF ^a^	10.82 ± 0.37	40.2 ± 0.25	5.8 ± 0.11	20.18 ± 0.31	54612 ± 169	Different strains, same process^b^	^11^
ASSF^a^	12.81 ± 0.25	44.4 ± 0.35	1.5 ± 0.17	24.68 ± 0.29	27641 ± 158		This study

^a^SSF: Conventional simultaneous saccharification and fermentation; ASSF: Adsorptive simultaneous saccharification and fermentation.

^b^Different strains, same process: Because the strains used in the reference could be not obtained in this study, we could only use the strains used in ASSF process, which was conducive to the comparison between different processes.

In addition, due to the importance of inulinase in the fermentation process, the effect of ASSF process on inulinase production by *A. niger* was also investigated. Table 1 shows that ASSF process could also promote inulinase production by *A. niger*. The specific reason might be that yeasts and pellets bind more closely in local scope, which alleviated the substrate inhibition of inulinase to some extent.

### The stability of pellets reusing technology

**Figure 6:**
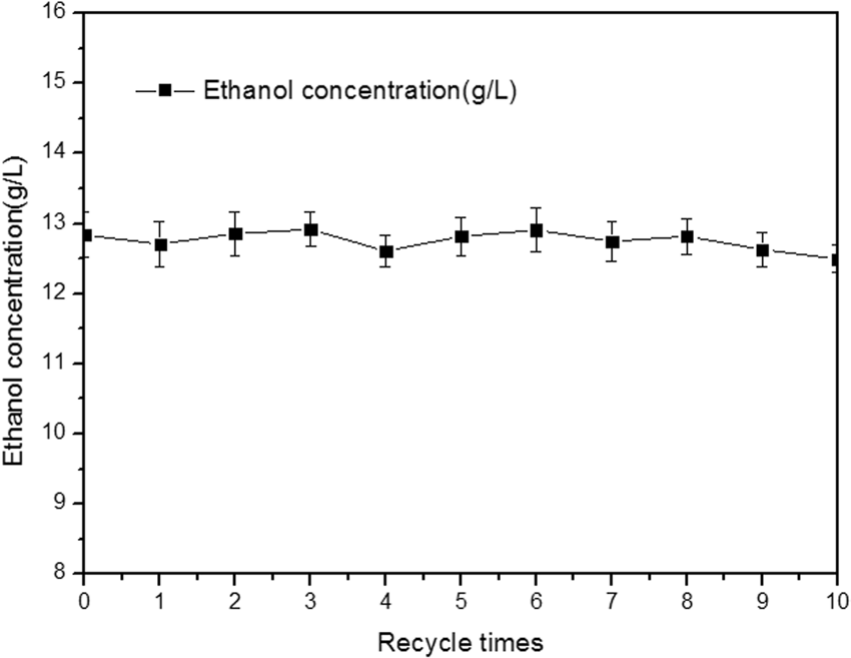
Effect of recycle times on the fermentation.

The effect of recycle times on the fermentation were investigated, and the results are shown in Fig. 6. After recycling 10 times, the ethanol production remained stable at a high level. Figure 5(c,d) shows the SEM of mycelial pellets recycling 1 and 10 times. Meanwhile, mycelia pellets have good adsorptive stability of yeast. The ASSF has the advantages of long service life, repeated use, easy operation and low cost. Therefore, carrier fermentation had a good prospect of industrial production.

### High ethanol tolerance test

High concentration fermentation of ethanol is a hot research theme in recent years. The key technology of this study is to obtain yeast with high osmotic pressure and high ethanol yield. It has become a research hotspot to improve the ethanol tolerance of yeast and to relieve the inhibitory effect of high ethanol concentration. The results showed that the ethanol tolerance of yeast depended not only on the inherent tolerance ability of yeast cells to different concentrations of ethanol, but also on the close relationship between the plasma membrane lipid compositions of yeast, nutritional status, environmental conditions, and supplementary mode of carbohydrate substrate,etc^28^. Critical ethanol concentration led to cleavage of plasma membrane phospholipids. With appropriate conditions of the plasma membrane of yeast, nutritional statusor environmental factors, the yeast can be resistant to ethanol toxicity. For example, with the temperature increasing, the phospholipid content of the plasma membrane in cell decreased rapidly to maintain the fluidity of the plasma membrane and cell viability^30^. Since *A. niger* is rich in lipoproteins which has been shown to improve the structure of the protoplasm membrane of yeast, the fermentation activity and ethanol tolerance of yeast are significantly improved. The combination of *A. niger* A-15 and *S. cerevisiae* C-15 could significantly improve the ethanol tolerance of *S. cerevisiae* C-15, as shown in Table 2.

**Table 2:** Ethanol tolerance of the strains.

Strain	Ethanol concentration∕%					
	12	14	16	18	19	20
*S.cerevisiae* C-15	+++	+	−	−	−	−
*A. niger* A-15	+++	++	−	−	−	−
Mixed pellets	+++	+++	+++	++	+	−

^*+++^Indicates that the strains grow well; ^++^indicates that the strains can grow; ^+^indicates that some strains can grow; ^−^indicates that the strains cannot grow.

As shown in Table 2, with the increase of ethanol volume fraction, the growth of *A. niger* C-15 and *S. cerevisiae* A-15 was gradually inhibited. However, mixed pellets could enhance the ethanol tolerance of yeast and moulds, and mycelial pellets could tolerate 19% (volume fraction) ethanol.

## Discussion

This technology had the following advantages: (1) It could convert the fermentable sugars from hydrolysate of inulin to ethanol simultaneously, preventing sugar accumulation which inhibited inulinase secretion, avoiding substrate inhibition, preventing bacteria pollution effectively, increasing ethanol yield. (2) Because yeast could not directly utilize inulin, inulin was first decomposed by inulinase produced by *A. niger*, so the whole fermentation process was a mixed fermentation of yeast and *A. niger*. The immobilization of yeast with *A. niger* did not incur any immobilization cost. Therefore, this technology could reduce the high cost of cell immobilization. (3) Mycelial pellets could be easily recovered from broth, and the technology has the advantages of long service life, repeated use, simple operation, low cost and easy to industrialize. (4) It is generally known that mycelium of *A. niger* is rich in lipoprotein which can improve yeast viability and ethanol tolerance. Due to the microenvironment change of *S. cerevisiae* after immobilization by pellets, the tolerance of *S. cerevisiae* to ethanol was enhanced. (5) Using JA as feedstock for ethanol fermentation is more economical. Compared with lignocellulosic biomass, JA neither requires the pretreatment with high technical difficulty and high sugar consumption, nor does it need expensive enzyme. Compared with starch materials, due to the low degree of polymerization of inulin, the hydrolysis of JA is easier to achieve than that of starch which needs high temperature liquefaction, and synchronous inulinase hydrolysis can be obtained fermentable sugar, simple process flow, lower energy consumption. Therefore, JA can be a green and economical option for ethanol production.

Mycelial pellets formed by *A. niger* A-15 were used to immobilize *S.cerevisiae* C-15, the yeast producing ethanol. The operation parameters, such as agitation speed, temperature and mixed proportion of strains were studied. The optimal adsorption 66.9% was obtained when speed was 80r/min, temperature was 40 °C and mixed proportion was 1:10. With JA flour as substrate, 12.8% (V/V) of ethanol was obtained after 48 h by simultaneous saccharification and fermentation using mycelial pellets. Considering the ethanol yield and residual sugar concentration, carrier fermentation is much more economical in terms of time and energy. Moreover, mycelial pellets could tolerate 19% (volume fraction) ethanol. The above results indicate that this new technology is feasible in producing ethanol.

In addition, this study mainly investigated the feasibility and advantages of ASSF as a new technology. Development of strains with high alcohol tolerance is a research hotspot in ethanol industry. More detailed examinations like AFM (Atomic Force Microscope) will be used to explain the improved ethanol tolerance of pellets in future research.

## Materials and Methods

### Microorganism and culture medium

The *A. niger* A-15 (CMCC98003) was stored in our laboratory. The *S. cerevisiae* C-15 was purchased from ANGEL YEAST CO., LTD.

Jerusalem artichoke was obtained from the local market in JiNan, China. The preparation of JA powder was to cut fresh JA into thin slices and air-dry them at 60 °C for 48 h, so as to reduce the water content to about 5%, and then ground to 60 mesh.

Seed medium(SM), in g/L: yeast extract, 10; peptone, 20; glucose, 20. The pH was adjusted to 7.0 by HCl or NaOH.

Medium for enzyme production (ME), in g/L: JA powder, 25; (NH_4_) _2_SO_4_, 5; peptone, 10; KH_2_PO_4_, 6; NaCl, 5; MgSO_4_·7H_2_O, 0.5; FeSO_4_·7H_2_O, 0.001. The pH was adjusted to 5.0 by HCl or NaOH.

Fermentation medium (FM), in g/L: JA powder, 200; peptone, 5; yeast extract, 5; (NH_4_) _2_SO_4,_ 2; KH_2_PO_4_ 2; corn syrup, 5. The pH was adjusted to 5.0 by HCl or NaOH.

YPD solid medium, in g/L: yeast extract, 10; peptone, 20; glucose, 20; agar, 20. Natural pH.

These media were autoclaved at 121 °C for 20 min.

### Cell culture

The culture of *S. cerevisiae* C-15 was harvested in 250 mL flask containing 100 mL seed medium with 5% of inoculum. The flask cultivated on a rotary shaker operating at 30 °C and 100 r/min for 30 h. The broth was centrifuged at 5000 r/min for 5 min, and then the cells were collected. Three replicates were carried out for each experiment.

*A. niger* A-15 was mixed with sterile water to form spore suspensions. The culture of mycelial pellets was grown in 250 ml flask containing 100 ml ME medium with 4% of inoculum. The flask was cultivated on a rotary shaker operating at 30 °C and 40 r/min for 30 h. The broth was centrifuged at 5000 r/min for 5 min, and mycelial pellets formed by *A. niger* A-15 were collected by filtration.

### Adsorption test

Yeast cells were suspended with 100 mL distilled water in a 250 mL Erlenmeyer flask, into which 5 g (wet weight) of pellet was added. The yeast cells were adsorbed at different temperatures, shaking speeds and different strains of the mixing ratio for 2 h. Then the cells were filtered by two-layer gauze, determined of the concentration of the cells that remained in the filtrate, and the adsorption rate was calculated with spectrophotometry.

1 $$\begin{array}{c}{\rm{Adsorption}}\,\text{rate}(R)=({{\rm{OD}}}_{600}{\rm{of}}\,{\rm{mixture}}\,{\rm{before}}\,{\rm{adsorption}}-{{\rm{OD}}}_{600}{\rm{of}}\,{\rm{mixture}}\,{\rm{after}}\,{\rm{adsorption}})\\ \,\,/{{\rm{OD}}}_{600}\,{\rm{of}}\,{\rm{mixture}}\,{\rm{before}}\,{\rm{adsorption}}\times 100 \% \end{array}$$

### Conventional simultaneous saccharification and fermentation

Yeast cells and *A. niger* Spore suspension were collected as described earlier, then mixed them in a certain proportion. The mixture of cells was grown in 500 mL flask containing 200 ml FM with 10% of inoculum(volume fraction). The flask was cultivated on a rotary shaker operating at 30 °C and 40 r/min for 48 h.

### Adsorptive simultaneous saccharification and fermentation

Yeast cells and *A. niger* spore suspension were collected as described earlier, then adsorbed in optimal temperature, agitation speed and mixed proportion of strains for 2 h. The mixture of cells was conducted in 500 mL flask containing 200 ml fermentation medium with 10% of inoculum(volume fraction). The flask was cultivated to produce ethanol on a rotary shaker operating at 30 °C and 40 r/min for 48 h.

### The stability of pellets reusing technology

Broth was centrifuged at 1000 r/min for 15 min, the collected mycelial cells were washed with normal saline and centrifuged at 1000 r/min for 5 min, and all mycelial pellets were transferred into fresh fermentation medium.

### High ethanol tolerance test

To ensure the ethanol tolerance of the mycelial pellets, these pellets were cultured in YPD solid medium supplemented with various concentrations of ethanol (12%, 14%, 16%, 18%, 19% and 20%). Pellets were inoculated at an initial cell density of 2 × 10^8^ cells/ml. Samples were collected, diluted, and plated on YPD solid medium. After incubation at 37 °C for 2 days, the colonies appearing on the plates were counted. Viability was expressed as a percentage of colony-forming units of the high ethanol treatment compared with control for each culture of the strains. Compared with the control group, the survival rate of strains was between 50% and 100%, which meant that the strain grew well. Compared with the control group, the survival rate of strains between 50% and 100% indicates that the strains grew well, the survival rate between 20% and 50% means that the strains can grow, while the survival rate between 0% and 20% means that the strains can not grow. The survival rate of strains was between 0% and 20%, which meant that it was difficult for the strain to grow.

### Analytical methods

The number of yeasts and fungi was determined by plate counting.

Ethanol concentration in the fermented medium was determined according to the methods given by Ge *et al*.^17^.

Residual sugars were determined by the 3, 5-dinitrosalicylic acid (DNS) method^31^. Total sugar was measured following the method reported previously^31^. Sample solution (1 mL), 50 ml of 2.8% H_2_SO_4_ was mixed and then heated at 100 °C to hydrolyze the carbohydrates. After 1 h, the solution was neutralized with 5 mol/L NaOH solution, and fixed capacity to 100 mL. The reducing sugar in the solution was determined by DNS method.

Inulinase activity was determined according to the method described by Susana M^17^. One unit of inulinase activity (U/mL) was defined as the amount of enzyme responsible for the production of 1 μmol of reducing sugar per minute at 55 °C and pH5.4.

2 $${\rm{Inulinase}}\,{\rm{activity}}={\rm{\rho }}(\text{glucose})\times {\rm{dilution}}\,{\rm{rate}}\times 1000/(180\times 10)$$

where 180 is the molecular weight of glucose (mg/L), 10 is the reaction time (min), 1000 is the unit conversion factor.

Scanning electron microscopy (SEM) was used to observe the structure and adsorption of mycelial pellets, and specific operation steps were determined according to the literature^32^.

3 $${\rm{The}}\,{\rm{yield}}\,{\rm{of}}\,{\rm{ethanol}}={\rm{m}}({\rm{Ethanol}}\,{\rm{production}})/m({\rm{the}}\,{\rm{decrease}}\,{\rm{of}}\,{\rm{total}}\,{\rm{sugar}}\,{\rm{in}}\,{\rm{broth}})$$

Chemical oxygen demand (COD) was determined by potassium dichromate method^31^.
